# Myelin Oligodendrocyte Glycoprotein Antibody Disease (MOGAD)-Monophasic Optic Neuritis and Epstein-Barr Virus (EBV): A Case Report of Rare Comorbid Diagnoses in an Adolescent From a Remote Greek Island

**DOI:** 10.7759/cureus.68946

**Published:** 2024-09-08

**Authors:** Efstratia-Maria Georgopoulou, Myrto Palkopoulou, Dimitrios Liakopoulos, Eleni Kerazi, Angelos-Michail Kalaentzis, Vanessa Barmparoussi, Michail Kokkinos, Anastasia Kaliontzoglou, Maria Anagnostouli

**Affiliations:** 1 Department of Neurology, General Hospital of Rhodes, Rhodes, GRC; 2 Department of Ophthalmology, General Hospital of Rhodes, Rhodes, GRC; 3 Research Unit of Radiology, 2nd Department of Radiology, School of Medicine, National and Kapodistrian University of Athens, Athens, GRC; 4 Multiple Sclerosis and Demyelinating Diseases Unit and Center of Expertise for Rare Demyelinating and Autoimmune Diseases of the Central Nervous System, 1st Department of Neurology, "Aeginition" University Hospital, School of Medicine, National and Kapodistrian University of Athens, Athens, GRC

**Keywords:** ebv, immunophenotype, mogad, multiple sclerosis, neuroimmunology, optic neuritis

## Abstract

A unique case of a female adolescent diagnosed with myelin oligodendrocyte glycoprotein (MOG) monophasic optic neuritis with Epstein-Barr virus (EBV) reactivation antibody profile on a remote Greek island is presented, highlighting the challenges of diagnosing rare conditions in rural settings and the importance of connecting centers of expertise with regional hospitals. The 16-year-old patient presented with progressive vision loss, headache, and retrobulbar pain in the right eye. Initial ophthalmological examinations showed decreased visual acuity and color vision deterioration. Magnetic resonance imaging (MRI) revealed optic perineuritis and edema. Cerebrospinal fluid (CSF) analysis excluded oligoclonal bands, and blood analysis was positive for both anti-MOG antibodies and EBV reactivation. Expert opinion and blood immunophenotyping confirmed the neuroimmunological condition. This case not only underscores the value of telemedicine in overcoming diagnostic challenges in rural settings but also contributes to the scientific discussion on neuroimmunological aspects and the potential role of EBV as an underlying factor in acquired demyelinating syndromes (ADS), beyond multiple sclerosis (MS).

## Introduction

Optic neuritis (ON) is a rare condition in adolescents, involving the inflammation of the optic nerve and perineural tissues. It leads to significant visual acuity loss, color vision deficits, retrobulbar pain, and optic disc edema. ON can be idiopathic or associated with rare neurological conditions such as myelin oligodendrocyte glycoprotein antibody disease (MOGAD). Myelin oligodendrocyte glycoprotein (MOG) is a protein on the myelin sheath of the neurons of the central nervous system. MOG antibodies are prevalent in up to one-third of pediatric acquired demyelinating syndromes (ADS), with an incidence of 0.87/100,000 [1]. Core clinical attacks, according to the latest diagnostic criteria, encompass ON, myelitis, or brain, brainstem, or cerebral syndrome, including acute disseminated encephalomyelitis (ADEM) [2]. Epstein-Barr virus (EBV) is a human herpesvirus that has been associated with multiple sclerosis (MS) pathophysiology and diagnosis [3]. Yet, the role of EBV in the specific setting of other neuroinflammatory diseases like MOGAD remains unknown. In the current report, we present an adolescent female case with an ON clinical course in favor of MOGAD and laboratory indications of the reactivation of previous EBV infection that stands among rare cases discussing EBV and MOGAD. Furthermore, this case is an example of a collaboration of centers of expertise with rural hospitals, minimizing the need for patient mobilization and emphasizing the cost-effectiveness of healthcare models while providing high-quality health advisory, rare disease diagnosis, follow-up, and treatment options.

## Case presentation

**Figure 1:**
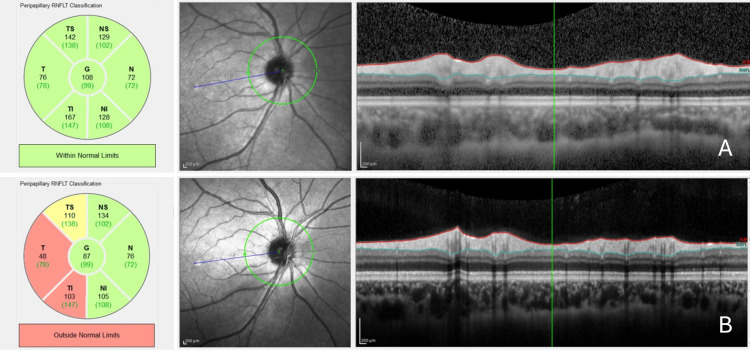
(A) OCT of the patient's right eye at presentation. (B) OCT of the patient's right eye at the six-month follow-up showing retinal nerve fiber layer atrophy ipsilateral to the optic nerve lesion. OCT: optical coherence tomography

Α 16-year-old female adolescent was referred to our Neurology Clinic from the hospital's Ophthalmology Department, due to reported progressive deterioration of her vision following retrobulbar pain in the right eye, during the last month. Her initial ophthalmological examination revealed a decrease in visual acuity 1/10 (decimal scale) and color vision deterioration, a normal appearance of the retinal vessels, and a halo surrounding the optic disc margins in the affected eye. The baseline optical coherence tomography (OCT) was within normal limits (Figure 1A). The ophthalmological examination of her left eye was unremarkable, and her neurological examination was otherwise normal. There was no reported history of recent or past significant infections. Her systemic history included skeletal disease (severe scoliosis). Birth was with cesarean delivery and non-complicated.

**Figure 2:**
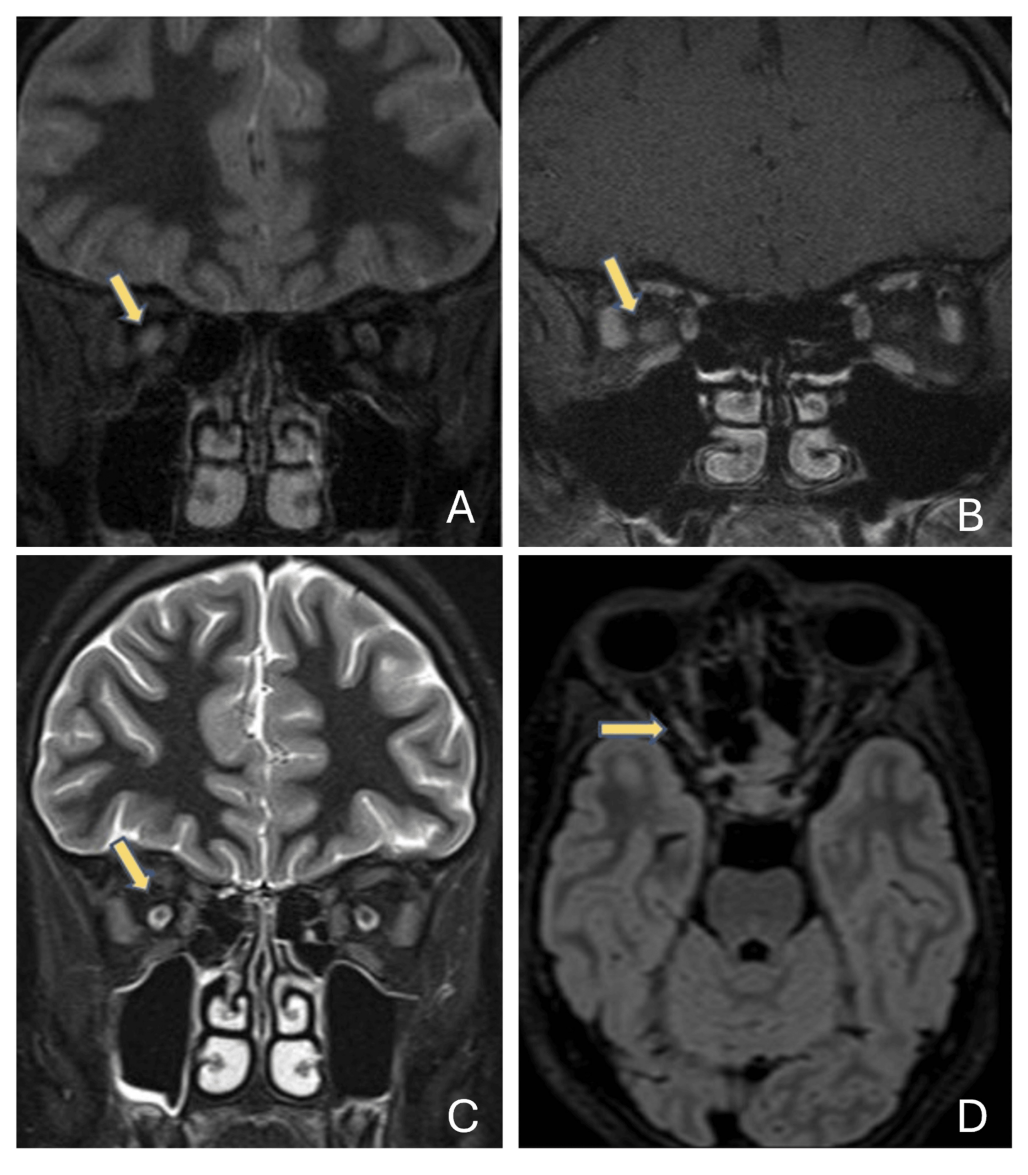
(A, B) PD coronal, T1 FS with Gd coronal MRI showing the unilateral involvement of the right optic nerve. Hyperintensity, enlargement, and strong enhancement of the intraorbital segment. (C, D) STIR coronal, FLAIR axial: subtle hyperintensity of the intraorbital and canalicular course of the right optic nerve without contrast enhancement. No signal changes of the optic chiasm or optic radiation. No evident disease regarding the brain parenchyma or spinal cord. PD: proton density; FS: fat suppression; Gd: gadolinium; MRI: magnetic resonance imaging; STIR: short tau inversion recovery; FLAIR: fluid-attenuated inversion recovery

Magnetic resonance imaging (MRI) of the orbits, brain, and cervical spine was performed, revealing a lesion with increased T2 signal in the middle part of the right optic nerve, which was enhanced with gadolinium contrast media (Figure 2A, 2B). Lumbar puncture with cerebrospinal fluid (CSF) analysis excluded the presence of oligoclonal bands (type 1). Blood analysis was negative for anti-aquaporin-4 antibodies but positive for anti-MOG antibodies. Anti-MOG titers were positive at the level of 1:5 (analysis with the enzyme-linked immunosorbent assay (ELISA) method).

The patient was admitted to our clinic for a five-day IV methylprednisolone treatment (1 g/day), with a satisfactory remission of symptoms during the third day of treatment and an almost complete remission at the one-month follow-up (9/10 visual acuity, normal color perception).

Further evaluation for autoimmune and viral conditions was declined by the patient's parents at that point, who consented two months later. The results were positive for EBV capsid antigen-antibody (VCA) IgG (titers 83.16 index value (IV), normal limits of the laboratory <1.0 IV) and IgM (1.17 IV, normal limits of the laboratory <1.0) antibodies (chemiluminescence immunoassay 98.3% sensitivity and 94.2% specificity for the double positivity for primary infection). The EBV nuclear antigen-1 (EBNA-1) and early antigen (EA) were positive as determined by ELISA. The patient was also negative for cytomegalovirus (CMV), and rheumatoid factor was not present. Anti-MOG titers were redefined with Western blot immunofluorescent IgG cell-based assay (IFA), and the results came back negative at that time point.

Ophthalmological examination results at the six-month follow-up were stable, except for signs of upper right quadrant atrophy of the right optic disc in the OCT examination (Figure 1B), consistent with the initial deficit. Follow-up MRIs of the brain and cervical spine showed increased T2 signal in the middle part of the right optic nerve and were otherwise normal (Figure 2C, 2D).

As highlighted above, the numerous diagnostic challenges of this case, including the availability of testing methods, sample transportation, and the financial and social issues typical of rural settings, pose additional challenges to the treating physicians, in parallel with the additional COVID-19 pandemic difficulties of that period. At that point, we contacted the Center of Expertise for Rare Demyelinating and Autoimmune Diseases of the Central Nervous System of the 1st Department of Neurology at "Aeginition" University Hospital in Athens in order to seek further guidance on diagnostic and treatment options. MRI of the thoracic and lumbar spine was suggested and carried out; however, it did not reveal any additional lesions. Blood immunophenotype analysis showed a slight increase in CD19+ cell count and a decrease in T-lymphocyte count, especially for CD4+. Nevertheless, the CD4+/CD8+ ratio was in favor of the total CD4+ count, supporting MOGAD diagnosis (Table 1). Anti-MOG antibody titers using cell-based Western blot-IFA technique in months 12 and 18 from disease onset revealed a marginally positive result.

**Table 1:** Immunophenotype results showing decreased T lymphocytes and slightly increased CD19+ B-cell counts. NK: natural killer cells; CAL: calibrated

Description/region	Result (% | CAL cells/uL)	Expected range (% | CAL cells/uL)
CD3+	64.7% L | 868.0 L	68.5-81.3 | 1500-1900
CD3+CD4+	37.2% L | 479.0 L	40-55 | 663-1477
CD3+CD8+	23.8% | 307.0 L	22-30 | 342-754
CD19+	22.7% H | 316	7.3-17.6 | 150-400
NK - CD3-CD(16+56)+	9.9% | 137	5.9-15.1 | 100-350
Ratio of CD4+/CD8+	1.6	1.4-2.6

The three-year follow-up of this case confirmed a monophasic disease course, no further relapses, and no other diseases or diagnoses so far.

## Discussion

Our case is particularly noteworthy and the first, to our knowledge, in the Caucasian population presenting MOGAD-monophasic ON in a patient with unique clinical features, including unilateral ON, an atypical OCT pattern for the disease, and the coexistence of EBV reactivation antibody profile. Additionally, the patient falls within an uncommon and underreported age group for the disease, adolescence, making the diagnosis even more challenging, especially in a rural setting.

Laboratory signs of EBV reactivation, indicated by detectable IgM and high IgG index values shortly after the initial episode in our case, are an emerging and controversial topic in MOGAD. On the one hand, MOGAD cases have been reported following infections, including EBV [4]. The simultaneous detection of IgM and IgG and EA and EBNA positivity without a clinical infection syndrome can occur during immune activation or EBV primary infection [5,6]. Regarding the interaction of EBV antibodies and MOG, EBNA-1 is considered to remain positive after acute infection and to interact with MOG, inducing demyelination [7]. A recent study in Japanese patients found EBV reactivation in both MOGAD and MS patients [8]. These findings are contradictory to the research that highlights the significance of EBV seronegative status in excluding MS [3,9]. On the other hand, EBV ON, which can also be monophasic and improve with corticosteroid treatment, has been described in adolescents, yet MOG antibody presence in these cases is not reported [10]. Other rare types of ON, such as glial fibrillary acidic protein (GFAP) ON and chronic relapsing inflammatory optic neuropathy (CRION), can also be considered; however, this case fails to align with the presentation features of any other type [11]. MOG antibody status in these cases remains an interesting field for further research.

Corticosteroid therapy and the potential for EBV reactivation due to immunosuppression cannot be excluded. However, the presence of EBV in this MOGAD patient represents an unexpected coexistence in the current literature. The relationship between corticosteroid treatment and prior EBV infection, particularly regarding the timing and duration of corticosteroid administration, is not well established in the context of pulse steroid treatment. However, it appears to be more thoroughly discussed in cases of chronic corticosteroid administration [12].

MOGAD-ON is the most common phenotype of MOGAD in the pediatric population after the age of 11 [11,13]. Severe visual acuity loss with good recovery, unless recurrent episodes occur, is a characteristic of MOGAD-ON [11]. MOGAD-ON often exhibits abnormalities in a fundoscopic examination, while perineural enhancement and longitudinal lesions are among its typical clinical features and are also present in our case [2,11]. OCT is a non-contact imaging modality, commonly used in ON cases, with a significant contribution to diagnosis. In our patient, OCT showed normal baseline results, unlike the retinal nerve fiber layer (RNFL) thickening in the acute phase that MOGAD produces. However, in children, MOGAD seems to present with some inconsistencies in clinical examination. Follow-up OCT indicated temporal RNFL thinning, an infrequent finding compared to the global atrophy seen in MOGAD-ON [14]. The same OCT findings could possibly indicate a first ON episode of MS. Overlapping clinical phenotype cohorts show that marginally positive MOGAD-Ab titers in classical relapsing MS cases can be disregarded, yet our patient presents a rather atypical clinical course for an MS clinical phenotype case [15]. Opposite to that, monophasic clinical course, negative oligoclonal bands, marginally positive MOG-Ab titers, lack of any relapse in extended follow-up, and fulfillment of core clinical attack criteria of the MOG diagnostic criteria also validated in large cohorts suggest MS is indeed a less prominent diagnosis [2,16].

When differentiating between ADS, cell cytometry arises as a potential tool. Research shows that there are distinct immune reactions and immune cell populations mediating inflammation in ADS clinical cases. Pathological studies in patients with MOGAD reveal that the lesions primarily contain CD4+ T-lymphocytes, which differs from the CD8+ T-lymphocyte predominance commonly found in MS lesions [17]. The most recent study on a large pediatric population from Horellou et al. aimed at examining immunophenotype differences between MOGAD children with and without relapse. Investigators observed a different response of T-regulatory lymphocytes between the relapsing and non-relapsing forms (T-regs decreased in relapsing and increased in non-relapsing) that could account for the different disease course [18]. CD19+ increased cell count, also evident in our patient immunophenotyping, confirms that an antibody-mediated process takes place, compatible with MOGAD. Despite the limitations imposed by the timing of the testing, immunophenotyping in our patient has significant utility in excluding acute EBV infection, which would be characterized by elevated CD4+ and CD8+ leukocyte counts [19]. We therefore propose that immunophenotyping could emerge as a valuable additional tool for differential diagnosis in these complex cases.

According to the latest systematic review on treatment in MOGAD, pediatric patients with a monophasic disease course are followed up in a watchful waiting manner, so our patient is currently under the suggested follow-up protocol and has not experienced a relapse during the three years following the first episode [20].

Despite contributing to the clinical and neuroimmunological dialogue on MOGAD, our case has some limitations. The MRI scanners used had a magnetic field strength of 1.5 Tesla, and different machines were used for neuroimaging. Variations in sample analysis methods, limited hospital resources, poor cooperation from the patient and parents, and the COVID-19 pandemic affected the timing and consistency of blood tests.

## Conclusions

This case highlights the diagnostic and therapeutic challenges associated with MOGAD-ON in an adolescent from a rural setting, emphasizing the importance of telemedicine and collaboration with centers of excellence. The patient's presentation, with potential EBV reactivation profile and MOG antibody positivity, adds to the ongoing debate about the role of EBV in neuroinflammatory autoimmune diseases. Continued investigation into MOG-Ab titers and repetitive MOG-Ab testing, immunophenotypic differences in MOGAD, and its ongoing expanding phenotypic spectrum will enhance understanding and improve patient care.
